# Hybrid Diagnostic Model for Improved COVID-19 Detection in Lung Radiographs Using Deep and Traditional Features

**DOI:** 10.3390/biomimetics8050406

**Published:** 2023-09-01

**Authors:** Imran Arshad Choudhry, Adnan N. Qureshi, Khursheed Aurangzeb, Saeed Iqbal, Musaed Alhussein

**Affiliations:** 1 Department of Computer Science, Faculty of Information Technology & Computer Science, University of Central Punjab, Lahore 54000, Pakistan; 2 Faculty of Arts, Society and Professional Studies, Newman University, Birmingham B32 3NT, UK; a.qureshi@staff.newman.ac.uk; 3 Department of Computer Engineering, College of Computer and Information Sciences, King Saud University, P.O. Box 51178, Riyadh 11543, Saudi Arabia; kaurangzeb@ksu.edu.sa (K.A.); musaed@ksu.edu.sa (M.A.)

**Keywords:** lung cancer, deep learning, convolutional neural network, batch normalisation, feature selection, classification, COVID-19

## Abstract

A recently discovered coronavirus (COVID-19) poses a major danger to human life and health across the planet. The most important step in managing and combating COVID-19 is to accurately screen and diagnose affected people. The imaging technology of lung X-ray is a useful imaging identification/detection approach among them. The help of such computer-aided machines and diagnoses to examine lung X-ray images of COVID-19 instances can give supplemental assessment ideas to specialists, easing their workload to some level. The novel concept of this study is a hybridized approach merging pertinent manual features with deep spatial features for the classification of COVID-19. Further, we employed traditional transfer learning techniques in this investigation, utilizing four different pre-trained CNN-based deep learning models, with the Inception model showing a reasonably accurate result and a diagnosis accuracy of 82.17%. We provide a successful diagnostic approach that blends deep characteristics with machine learning classification to further increase clinical performance. It employs a complete diagnostic model. Two datasets were used to test the suggested approach, and it did quite well on several of them. On 1102 lung X-ray scans, the model was originally evaluated. The results of the experiments indicate that the suggested SVM model has a diagnostic accuracy of 95.57%. When compared to the Xception model’s baseline, the diagnostic accuracy had risen by 17.58 percent. The sensitivity, specificity, and AUC of the proposed models were 95.37 percent, 95.39%, and 95.77%, respectively. To show the adaptability of our approach, we also verified our proposed model on other datasets. Finally, we arrived at results that were conclusive. When compared to research of a comparable kind, our suggested CNN model has a greater accuracy of classification and diagnostic effectiveness.

## 1. Introduction

Following its discovery in December 2019 in Wuhan, Hubei Province, China, COVID-19 has quickly spread since then. COVID-19 was deemed a worldwide medical emergency by the World Health Organization (WHO) [1]. The overall majority of patients being probed and tested worldwide has topped 6 million, and more than 300,000 people have died as a result, posing a serious threat to people’s health across the globe. The SARS-CoV-2 (Severe Acute Respiratory Syndrome Coronavirus 2) virus is what causes COVID-19 [2].

The sickness caused by SARS-CoV-2 was dubbed COVID-19 by the World Health Organization (WHO) in February 2020. During this time, the WHO advised that the best method of preventing viral illness propagation was to encourage self-isolation and trace close connections as soon as possible. The proper diagnosis and screening of COVID-19 patients allows for affected people to receive prompt treatment, as well as successfully preventing the malevolent virus from spreading further. Detecting and diagnosing COVID-19 is now the most challenging task [3].

Research has shown that imaging methods (such as X-ray or CT) are more sensitive than RT-PCR for diagnostic and monitoring purposes of COVID-19 [4]. Even while real-time X-ray imaging may significantly allow faster disease monitoring, Computed Tomography (CT) scans frequently take more computational time than X-ray imaging. Additionally, some less-urbanized places might not have enough adequate CT scanners. Most convalescent patient clinics, hospitals, and other institutions have extended and deployed lung X-ray instruments and equipment as essential scanning equipment due to their low cost and ease of use. X-ray imaging, as opposed to CT imaging, is the most frequent and commonly utilized medical imaging technique, and it is essential in clinical nursing and epidemiological research. As a result, the research object for this study was chest X-ray scans. When interpreting X-ray images, however, medical practitioners, radiologists, and specialists mostly interpret and explicate images based on personal clinical experience [5]. Different specialists and radiologists usually have different interpretations of the unchanged image. Further, the status of the unchanged image at different times is not constant, resulting in varied findings. Furthermore, the effort of image interpretation is enormous, and clinicians are prone to misinterpretation owing to exhaustion. As a result, a computer-aided diagnostic system is urgently needed to assist radiologists in interpreting images quickly and more precisely [6].

Medical scanning detection and monitoring are progressively using artificial intelligence. Deep learning, specifically convolutional neural networks (CNNs), have indeed surpassed individual people in the area of image processing, computer vision, and cognition [7]. Rajpurkar et al. [7] proposed a concept for CheXnet as a pulmonary and pneumonia classification algorithm. The findings presented by the author of the training methods to detect 14 lung ailments using the ChestX-ray14 dataset [8] gave results which were better than a conventional radiologist’s diagnosis. There have been several previous research reports on COVID-19: Wang et al. [9] introduced a unique CNN deep learning-based model framework (COVID-net) as well as a wider dataset (COVIDx (containing 13,800 lungs X-ray radiological images)). The goal is to categorize lung X-rays into three categories: pneumonia, normal, and COVID-19. COVID-19 had a diagnosis accuracy of 92.4%, according to the findings. Karar et al. [10] examined three classic deep learning-based categorization frameworks and used a pre-trained CNN model to identify between normal and COVID-19 using the ImageNet dataset [11]. They used a diminutive dataset of just 50 lung X-ray images in the experiment, 25 were of which were from healthy individual patients (negative COVID-19), and the other 25 were from COVID-19-positive patients. VGG19 and DenseNet performed similarly in the author’s model, with F1-Scores of 89.12 and 91.76 for normal and COVID-19, respectively. Farooq et al. [12] provided a refined ResNet50 architecture that classified lung X-rays as COVID-19 and Normal, respiratory disease, or highly contagious pneumonia.

The researchers state that their method is more accurate than COVID-net [9]. Apostolopoulos et al. [13] conducted extensive tests using transfer learning on pre-trained CNN models. Finally, the scientists discovered that VGG19 surpasses other deep learning-based CNNs in terms of efficiency. Narin et al. [14] employed transfer learning to identify COVID-19 and constructed three distinct deep CNN models, including ResNet50, InceptionV3, and InceptionResNetV2. While various techniques for coronavirus recognition have been devised and deployed, there is always potential for performance improvement for diverse datasets.

Despite the research described above, the review found that deep learning or other approaches for diagnosing and screening COVID-19 in X-rays are rarely used. As a result, our objective is to develop an effective mix of deep learning-based automated features and machine learning categorization methods to aid medical experts and radiologists in more reliably diagnosing COVID-19 in X-ray images. This study addresses the suggested system’s inability to ensure computational complexity while enhancing the accuracy rate. The following are the primary contributions of this work:

1. Initially, transfer learning is utilized to solve the generalization error produced by deep learning’s restricted amount of training images. We created a dataset of 1102 lung X-ray images of healthy and normal patients and COVID-19-positive patients, then arbitrarily and randomly separated the validation, testing, and training sets due to the unavailability of a publicly available COVID-19 dataset. The ImageNet dataset was used to pre-train four familiar Convolutional Neural Network models such as Xception, InceptionV3, VGG16, and DenseNet121. Their achievement was measured using a testing set of 298 lung X-ray images. Our top model (Proposed Model) has a 94.01% accuracy rate.
2. We employed a method for obtaining manual and automated features from deep convolutional neural networks which are automated. This strategy avoids the need for sophisticated feature extraction operations by eliminating the use of traditional manual methods. Gating factor features may be extracted directly from four pre-trained depth models using this approach. COVID-19-positive patients are examined and screened using four typical machine learning algorithms after barrier characteristics are extracted.
3. Extensive testing has revealed that each deep model performs admirably on a variety of classifiers. The top model’s accuracy is as high as 99.57%. It is worth noting that our top model also performs well on a different dataset.

Artificial intelligence (AI) and machine learning (ML) are currently being used in the field of medicine to gather data from many sources, assisting in the prediction and diagnosis of diseases that are otherwise difficult to diagnose. According to numerous studies, AI has proven to execute some jobs better than humans, as mentioned by Davenport and Kalakota [15], and this is especially true in fields such as medical imaging, as highlighted by Lee et al. [16]. AI is a helpful addition to human labor, increasing the effectiveness of many tasks. Notably, as described by Lee et al. (2017), recent developments in deep learning methods designed for medical image processing have produced encouraging results across a number of applications, including segmentation and registration. Among these methods, deep neural networks (DNNs), particularly convolutional neural networks (CNNs), have drawn a lot of interest as a potential approach to problems in the field of medical imaging.

This paper acknowledges the necessity of utilizing machine learning (ML) and artificial intelligence (AI) to overcome these issues. Although COVID-19 detection is the immediate emphasis, this research’s wider significance is clear. We have the ability to transform diagnostic procedures for a range of medical diseases by implementing AI into medical imaging. The accomplishment of this project not only improves COVID-19 diagnosis but also lays the groundwork for the use of AI in other medical specialties. Additionally, AI-driven diagnostic tools can decrease accessibility gaps in healthcare, especially in areas with poor access to specialized medical knowledge. AI-enabled diagnostics can help local healthcare providers in remote and underdeveloped locations make accurate decisions and improve patient outcomes. Therefore, this technology’s effects go far beyond the current pandemic. This study also addresses the inherent drawbacks of human-centric diagnosis, such as subjectivity, errors brought on by weariness, and variances in skill. We want to improve patient care by implementing a computer-aided diagnostic system that will both increase radiologists’ abilities and reduce the possibility of incorrect diagnosis. Our study aims to improve COVID-19 detection with cutting-edge AI methods. The ramifications, meanwhile, go beyond the need for an early pandemic response. We seek to transform the diagnostic environment, improve accessibility to correct healthcare, and usher in a new era of medical technology with ramifications across various medical problems by pioneering AI applications in medical imaging.

The remainder of the paper is structured as follows: in Section 2, this investigation’s methodology is described. The experimental process is introduced in Section 3. The experimental results are discussed in Section 4. Finally, the study is summarized in the final section.

## 2. Background

**Figure 1:**
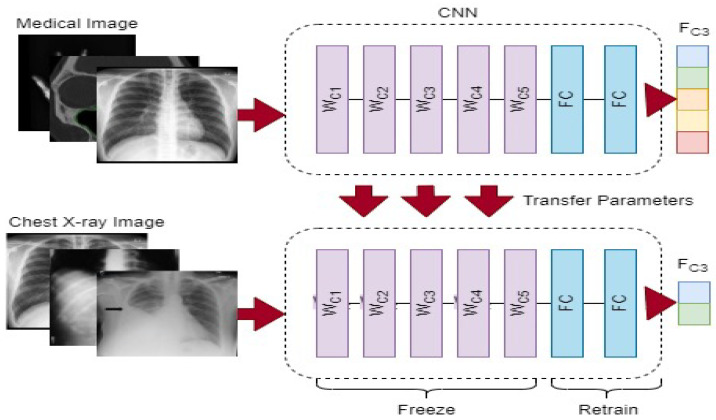
Convolutional neural network (CNN) model flowchart for transfer learning fine-tuning.

It could be challenging to gather a large dataset in the field of medical and radiological imaging. Several approaches are incapable of producing superior outcomes in the few radiological images that have been recognized as COVID-19 because of the low number of these images [17,18,19]. A convolutional neural network (CNN) model frequently needs a lot of annotated images to train, which might be problematic since it can find out the real proportion of the source images and can potentially result in overfitting. To address these issues, we first employ a commonly utilized method known as transfer learning (the use of a model that has been pre-trained on a large labeled dataset for a new task), as illustrated in Figure 1.

We would require a large amount of content, powerful computer power, and a great amount of patience to build a neural network from scratch, making it an unfeasible task. Instead, we modified a deep learning network model’s settings to fit the demands of the new task at hand. Frequently, the upper part of the network architecture retains only basic characteristics. The model gradually comprehends more exact sequences for training as its size increases. We chose to only fine-tune the final or penultimate layer(s) of the convolutional neural network (CNN) in our technique as a result of the restricted number of COVID-19 lung X-ray images. This required isolating the nearby convolutional layer so that the tailored FC layer could be trained in isolation once the fully connected layer from the top of the pre-trained model was removed and replaced. VGG16 [20], InceptionV3 [21], DenseNet121 [22] and Xception [23] were used to analyze the quality of four regularly used models. Tables 1 and 2 shows the entire features of the four pre-trained networks used in this investigation, including their input size, number of layers, and number of parameters. We will explore the architecture of these models quickly below.

Simonyan et al. [20] investigated VGG16. The model participated in the 2014 ImageNet Large Scale Visual Recognition Challenge (ILSVRC2014), winning first place. Compared to AlexNet, it used a smaller convolution kernel and fewer features, and the categorization performance is markedly better. The two variants of this CNN architecture are VGG16 and VGG19. For instance, VGG19 has additional levels than VGG16, as well as more features and larger latencies.

In 2014, InceptionV3 [21] came in first on GoogLeNet, with a top-five accuracy of 93.3%. A bigger two-dimensional convolution is divided into two tiny one-dimensional convolutions by the system. It not only cuts down on the number of features, but it also ramps up computation and prevents generalization errors. InceptionV3’s architecture stresses the relevance of memory management and the model’s computational capabilities.

Xception [23] takes the Inception approach to its logical conclusion. It is assumed that inter and spatial correlations may be distinguished. Additionally, using the ImageNet dataset, the classification rate is somewhat better than InceptionV3. On large-scale image data sets, using the same number of parameters can enhance efficiency.

In recent years, ResNet50 [24] has become a highly popular convolutional neural network architecture. It attained first place in the ILSVRC2015 competition. Its novel remnant artifact accepts a more straightforward gradient movement and even more adequate training.

The most recent network design is DenseNet121 [22]. It took first place in the 2017 ImageNet competition. It makes use of characteristics in order to obtain better outcomes with lower dimensionality. It can link all levels directly if the maximal information flow between layers in the network is ensured.

Rehman et al. [25] proposed a unique paradigm for COVID-19 classification using a lung X-ray modality to diagnose 15 different forms of lung illnesses, including COVID-19. In the proposed methodology, two-way categorization is performed. Initially, a deep learning-based CNN model with a softmax classifier is used. Second, a suggested CNN’s fully connected layer with deep extracting features is used to apply transfer learning. The traditional machine learning classification techniques are supplied with deep spatial features.

Allioui et al. [26] proposed a novel technique for the enhancement of COVID-19 CT image segmentation. The effectiveness of automated image classification is increased by the introduction of collaborative bots in mask derivation. Therefore, in order to reduce the need for human long-term mask separation and improve medical image classification approaches, they provide a novel mask separation technique based on multi-agent deep reinforcement learning (DRL). In order to address problems with mask extraction, a DRL-based technique is presented. The mask sensor may choose masks from the image under study using this novel technique, which modifies the Deep Q-Network. In order to obtain the physical characteristics of COVID-19-contaminated regions and deliver an appropriate medical assessment while streamlining the pathogenic diagnostic procedure and cutting down on effort, they applied DRL mask extraction-based approaches based on COVID-19 computed tomography (CT) images.

Saeed et al. [27] suggested numerous computational models for evaluation.The complex fuzzy hyper-soft (CFHS) set, which is a creation of the complicated fuzzy (CF) set and the hyper-soft set, is a unique agile fuzzy-like configuration that serves as the foundation for the theoretical formulation presented in this article (an extension of the soft set). In order to address ambiguity, antagonism, and inferiority of data, the basic theory of fuzzy hyper-soft is first created, which takes into account the amplitude term (A-term) and the phase term (P-term) of the imaginary figures concurrently.

Abdulkareem et al. [28] proposed to provide a multidimensional examination framework (MEF) based on integrated multi-criteria decision-making (MCDM) techniques for prioritizing individuals seriously affected by COVID-19. The recommended methodology for the multidimensional examination framework contained many multivariate evaluation parameters, including demographic, analytical results, vital signs, feelings, and chronic illnesses, in contrast to the current research, which only addressed one aspect of the evaluation variables.

Fang et al. [29] proposed a unique framework for the COVID-19 classification model using a combined computed tomography and X-ray dataset. This study uses a combined dataset of CT and X-ray images to develop a unique coronavirus pulmonary diagnosis algorithm. An evolutionary area improvement approach is used to improve the derived features in order to address the issue of feature resemblance between COVID-19 and lung illnesses. Additionally, the deep network built using dense and residual blocks has undergone training and testing.

## 3. Proposed Method

**Figure 2:**
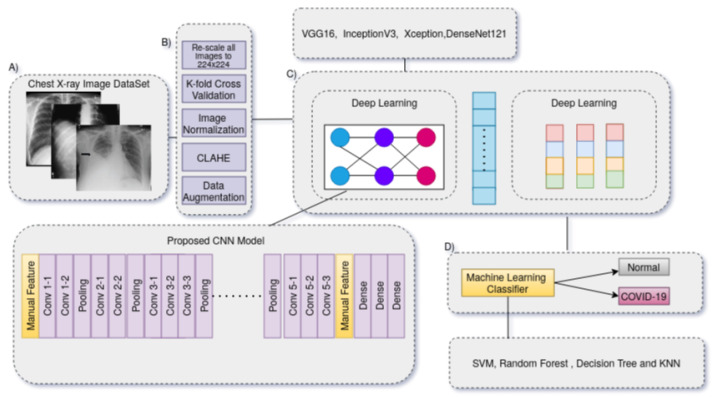
COVID-19 recognition system: (**A**) We utilized lung X-ray, (**B**) applied different preprocessing algorithms, (**C**) reloading transfer deep learning models such as VGG-16, Inception, DenseNet, and Xception over pre-compiled images in the image datasets, (**D**) and the features of different pre-trained models are forwarded to different conventional machine learning algorithms such as SVM, Random Forest, Decision Tree and KNN.

We present a technique for automatically diagnosing COVID-19 in X-ray images by combining deep CNN features and manually extracted features with machine-learning classification algorithms. Figure 1 depicts the suggested method’s flow. The conceptual approach consists of three primary phases that must be completed in order to complete the COVID-19 diagnostic procedure. The strategy discovered in this study depicted in Figure 2 reduces lengthy pre-processing procedures and improves the generalization tolerance of CNN architectures. It usually makes the technique more resistant to distortion, artifacts and fluctuations in the input image during the feature collection process. As a consequence, we only utilized two straightforward pre-processing steps and data up-sampling approaches for training the CNN model. Because the images in the given dataset may have been collected from various sensors, the image-capturing settings are also diverse, and that each image seems to have a distinct pixel size. As a result, there are considerable fluctuations in the image’s strength and shape. We then scaled all of the images to 224 by 224 pixels.

The research provided in this paper focuses on several pre-processing strategies to improve model training for feature extraction capabilities. A radiography image can become complicated for a variety of reasons, the most common of which being contrast fluctuation and the patients’ sudden movements. The values added to the pixels of an image that causes a change in the image information is known as noise. The goal of pre-processing is to bring the dataset images to their full potential. We started by resizing the dataset to a 224 by 224 input size, then applied de-noising, edge detection, and histogram equalization on every image in succession before submitting it to the learning algorithm. We utilized a Gaussian filter—a non-uniform low-pass filter based on a 2D convolutional filter—to eliminate noise from the dataset during de-noising. It smooths and preserves edges by using the weighted average of nearby pixels in radiography [30].

Edge detection methods aid in the differentiation of features and are one of the most often-employed operations [31]. Auto-canny depicted in Equation (1) has been utilized to identify edges in radiological images [32]. Because the edges carry the majority of the image’s information, edge detection is a crucial part of pre-processing. Auto-canny carries out four primary functions. The border improvement amplifier decreases the distortion in the radio-graphic images first, then evaluates the slope to identify the derivative’s maximum, which is perceived as the position of the edge pixel in the radio-graphs. It also employs non-maximum suppression and edge detection delay, which disables weak pixels. CLAHE (contrast limited adaptive histogram equalization) is being used to boost the distinction of the dataset by enhancing the fine features of the radiographs. As a result, even in places that are darker or brighter than the rest of the image, local features can be enhanced [33]. The goal of adopting image processing techniques in the study is to see if they may help doctors by speeding up the training and diagnosing process [34].

(1) $$\begin{array}{cc} N_{ij} & =\frac{1}{2\pi \sigma^{2}}\exp\left(\frac{{\left(i-\left(k+1\right)\right)}^{2}+\left(j-{\left(k+1\right)}^{2}\right)}{2\sigma^{2}}\right)\\ \; & =1\leq i,j\leq (2k+1)\\ \left|G\right| & =\sqrt{I_{x}^{2}+I_{y}^{2}}\\ \theta (x,y) & =\arctan(\frac{I_{y}}{I_{x}}) \end{array}$$

A sizeable amount of the lung X-ray image dataset images come from various pieces of acquisition equipment, each of which uses different device settings. As a result, there are noticeable differences in pixel intensity between the images. In order to ensure that all images lie inside the [−1, 1] range, we normalize the pixel intensity for each image. The benefit of this normalization method is that it lessens the model’s sensitivity to minute changes in weights, allowing for smoother tuning.

**Figure 3:**
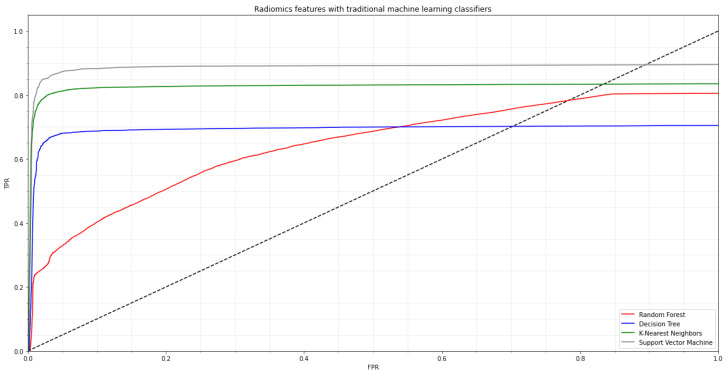
The ROC curve for the COVID-19 training dataset by employing the traditional machine learning classifiers such as random forest (RF), decision tree (DT), k-nearest neighbor (K-NN) and support vector machine (SVM).

The basic technique for the COVID-19 identification system is shown in Figure 3. To learn exclusionary and practical characteristic models, it is necessary to reload a transfer DL approach (VGG-16, Inception, DenseNet, and Xception) over precompiled images in the image datasets. The process for building the data store is briefly discussed first. The specifications of the proposed model are then detailed, such as the fundamental design, the selected approach’s training scheme, and the suggested pre-processing techniques. This process also involves normalization, image down-scaling, contrast improvement, and data enrichment. Overfitting results from learning more characteristics than necessary as the model’s network gets more complex. Data enrichment was utilized to prevent distorted prediction results after the COVID-19 dataset was split into three sets that are mutually exclusive (e.g., the training, validation, and evaluation sets) to address the overfitting problem due to the lack of training images. For each image in the collection, supplemented images with appropriate masks for orientation, mirroring, tilting, and resizing are produced. The capability for screening and prevention is hampered by the inadequacy of raw COVID-19 X-ray images generated by an electronic detector. Image-enhancing methods should be applied to raise the caliber of COVID-19 X-ray images. Additionally, reducing the generalization error and training duration of convolutional neural networks (CNNs) by using pre-processed images rather than raw image data. In order to fix the poor resolution of the COVID-19 X-ray image before entering it into the suggested system, a suitable image-enhancing approach was developed. Small features, textures, and brightness adjustments in the COVID-19 X-ray image were enhanced utilizing reactive contrast enhancement depending on shifting the brightness levels in the input image. This method reallocated the frequency distribution of the input image and used the appearance as an input to create an enhanced image. As a result, this method increases the contrast enhancement of the image while simultaneously improving the transparency of the borders and contours in each area of the image. The characteristics of image capturing frequently change since some images have low-resolution sizes and all of the images must be resized because the images in the dataset are drawn from separate datasets and may also originate from different X-ray machines in various radiological laboratories and hospitals. As a result, the image’s intensity and scale are significantly altered. Additionally, the Kaggel dataset only contains grayscale images; in order to create an RGB image, we should repeat the image three times. It is quite likely that the majority of the images in the CXR image collection came from different acquisition equipment, each of which has its unique needs. Each image’s component brightness might differ hugely, therefore to guarantee that the data are within certain noise and ranges is eliminated, the pixel intensities of all images are normalized around [−1, 1]. The advantage of normalization is that it makes the model less sensitive to minute changes in weights, which facilitates optimization.

Overfitting occurs as the amount of trainable parameters increases along with the complexity of the CNN model. We employed data augmentation (rotation, flipping horizontally and vertically, shearing, padding, scaling, and zoom) to tackle the overfitting problem caused by the minimal number of training images in this scenario. We randomly rotated images by 30 degrees and randomly zoomed them by 20% [35].

Deep learning model evaluation can be a labor- and time-intensive operation. The dataset is typically divided into different training and testing ratios. Cross validation, also known as the k-fold validation procedure, is one of the most widely used statistical techniques for evaluating the effectiveness of deep learning models and reducing overfitting. We used the k-fold cross-validation strategy in our effort to improve our machine learning model’s predictive capacity. To ensure that there is no input bias, this entails thoroughly randomizing the dataset within the k-fold cross-validation technique. The dataset is additionally split into k identically sized parts with no overlap.

The fundamental architecture of the suggested system is based on the convolution layer, residual components (extracted from ResNet [24]), and manual feature grafting. The most challenging task when utilizing deep learning models is determining the enormous number of features and hyper-parameters (e.g., epoch value, number batch size, momentum, learning rate, optimizers, number of frozen layers, number of layers, etc.). The investigation is conducted on how different hyper-parameter values affect how well the suggested systems work. In this part, we explore a possible solution based on a proposed model COVID-Net in great depth. The residual unit is compensated by a conventional layer with a skipped connection. By connecting the input channel to the layer’s outcome, the skip connection allows the data to traverse the network. As a result, weights are temporarily allocated at random to the newly updated layers. The basic technique for developing neural network models, back-propagation, is then used to modify all model parameters throughout learning. We adopted this tendency of adding extra fully connected (FC) layers at the conclusion of the proposed model after experiments utilizing the proposed CNN model without adding an additional FC layer produced extremely poor results. Two fresh FC layers with a capacity of 512 and one FC layer with a capacity of 3 were added. The class labels are inserted after the modified FC layer and before the Relu layer, which was also changed with a new leakyRelu layer. The network requires more FC layers while handling small datasets than when handling bigger datasets. The completely linked layer connects every neuron from the previous layer to every additional neuron in the following layer, and each number helps anticipate whether a value matches a certain class. The activation function—softmax—then determines the class scores using the output of the last FC layer. One of the most popular CNN activation functions is leakyRelu. The fact that adding a single FC layer requires a lot of computation is its main downside. Between the initial FC layer and the leakyRelu layer, which were likewise rebuilt by new FC layers and leakyRelu layers, respectively, we added three more FC layers. The initial FC layer is 2048, the second FC layer has 1024, and the final is 3 in size. Because the network performance of the model happens when a model absorbs the training data extraordinarily well but may not generalize well to additional testing data, we employ batch normalization because it is good at preventing this. The likelihood of encountering this issue rises when the training dataset is minimal, as it was in our work. This is a typical problem in deep learning models. Because several phases in such procedures entail some amount of variation, CNN methods always yield outputs with some degree of unpredictability. So, using ensemble machine learning is one technique to optimize the effectiveness of CNN algorithms. One of the definitions of ensemble employed here is to repeat the system N-times with the same network settings (epochs, number of layers, batch size, activation function, and optimizer). The numerous ensemble is how we propose to use stacking generalization in our work. It involves running numerous training sets on the very same model.

The transfer learning experiment of this study’s fine-tuning and ensemble machine learning approach did not produce any remarkable results. We provide a unique representation mechanism for convolutional neural network features to improve the model’s generalizability. This method uses VGG16, Inception-V3, DenseNet-121, and Xception, four pre-trained CNN models, as feature extractors. Encoding is applied to the first input image to create the CNN and manual feature vector for the image descriptor. The automated characteristics derived from each model are then retrieved once this encoded feature vector is generated for each model. When compared to retraining the CNN model after fine-tuning, these recovered bottleneck features offer a lower-dimensional vector, which significantly reduces model training time. Additionally, we manually retrieved other statistical characteristics from the NIH X-ray dataset, such as the sum of variance (SoV), sum of entropy (SoEnt), difference of entropy (DoEnt), entropy (Ent), energy (E), sum of average (SoA), and difference of variance (DoV). As shown in Table 1, the requisite pre-trained techniques were used to store these pertinent features in separate arrays for later use. The size of pixel intensity values in an image is quantified by energy. High-energy values in the context of medical image analysis may correspond to regions with distinct edges or abrupt transitions, which can be important for identifying characteristics such as boundaries or lesions.

The variability of pixel intensity values in an image is measured by the sum of variance. It can reflect the overall texture and structural variety of the image in medical image analysis. This characteristic can be used to record variations in lung tissue density that may be a sign of anomalies such as fluid accumulation or lesions.

The complexity or randomness of the pixel intensity distribution is measured by the sum of entropy. Entropy frequently reflects the degree of detail and texture in medical imaging. Inconsistent patterns or heterogeneous regions may be suggested by higher entropy, which may be important for determining disease states.

A measurement of the unpredictability or uncertainty in pixel intensity levels is called entropy. Higher entropy in medical imaging may signal the presence of intricate textures or erratic patterns, which may be linked to specific anomalies or abnormalities.

The difference of entropy calculates the difference in entropy between several areas of the image. This feature can aid in the detection of sudden shifts between various tissue types or structural elements in medical image analysis, which may be a sign of alterations brought on by a disease.

The image’s average pixel intensity levels are determined by the sum of averages. It can aid in capturing the general brightness or density of tissue regions during medical imaging. Aspects divergent from the usual areas of interest, e.g., lesions or anomalies, may stand out.

The variance of pixel intensity differences between neighboring pixels is calculated using the difference of variance. This capability is excellent for capturing small changes in pixel brightness that may be brought on by little details, noise, or subtle patterns. The varied texture, structure, and intensity changes seen in medical images, especially X-ray images, are meticulously captured by the manual features. Each component offers distinct insights into various aspects of the image, and all of them together help distinguish between normal and aberrant patterns. Machine learning algorithms can increase the diagnosis process’ accuracy by studying these features and learning to identify the particular patterns connected to COVID-19 instances.

The decision to use a particular set of manual features for the analysis of X-ray images, particularly in the context of COVID-19 diagnosis, is made based on how well those features can identify the essential elements of lung image abnormalities, textures, and structural irregularities. The rationale for choosing these manual features and their significance for COVID-19 diagnosis are as follows:

Changes in lung tissue density, such as ground-glass opacities and consolidations, are frequently brought on by COVID-19. Pixel intensities alter as a result of these adjustments. SoV can quantify the variety of these intensity variations, assisting in the distinction between areas that are normal and those that are affected by COVID-19. Higher SoV levels could be a sign of complex lung problems. In lung images, COVID-19-related anomalies frequently introduce complicated textures. These textures’ richness and irregularity are beautifully captured by SoEnt. Higher SoEnt areas could signify the presence of places with a variety of patterns, possibly indicating COVID-19-affected areas. Changes in COVID-19 can affect the local fluctuations in pixel intensity. DoV records the variance differences between neighboring pixels, which are susceptible to minute structural or textural alterations. This feature may be useful for locating COVID-19-related localized anomalies. These manual factors have been selected because they may be able to identify particular visual traits connected to COVID-19-related anomalies in X-ray images. Machine learning models can better learn to distinguish between typical and COVID-19-affected patients by including these features in the diagnosis procedure. The chosen features help to assist an accurate and timely diagnosis of COVID-19 by collectively shedding light on the many patterns and anomalies that are indicative of lung problems.

Initially, we store each model’s checkpoint feature and then feed the automated and manual features into four different machine learning classifiers (SVM [36], random forest [37], decision tree [38] and K-nearest neighbors (KNN) [39]). Finally, COVID-19 cases and normal cases were assigned to all X-ray images.

## 4. Experiment

As a novel disease, COVID-19 lacks a suitable dataset for this study project. We combined and modified three publicly accessible datasets to avoid this restriction. Our research was limited to COVID-19-related anterior-posterior (AP) and posterior-anterior (PA) lung X-ray images. Up until 15 September 2020, individuals with COVID-19 and other illnesses, such as Middle East Respiratory Syndrome (MERS), Severe Acute Respiratory Syndrome (SARS), and Acute Respiratory Distress Syndrome (ARDS), provided over 657 X-ray images for this combined dataset.

In our investigation, 500 X-ray images from patients with COVID-19 were chosen for analysis. The COVID-19 Lung X-ray Initiative, shown in Figure 1, supplied an additional 37 lung X-ray images from COVID-19 patients to the second dataset. We used resampling, specifically random under-sampling, to address the data imbalance problem. Using this method, instances from the classification model were gradually removed until a more fair distribution within the dataset was attained. In addition, a random sample of 565 regular chest X-ray images was taken from the Kaggle dataset.

We compiled a thorough set of 1102 chest X-ray images by combining these three different public datasets. This collection includes 537 instances of COVID-19 and 565 images showing typical occurrences. The images were randomly divided into testing (15%), validation (15%), and training (70%) groups in order to achieve objective testing and validation. The inclusion of several photographs from the same person in the training or test sets is guaranteed by this random distribution technique. The validation set will make up 15% of the training set during the training phase.

### 4.1. Evaluation Metrics

The chest X-ray image was evaluated using a variety of assessment indicators in this investigation to assess the effectiveness of both the transfer learning approach and our suggested technique. The following are the evaluation indices depicted in Equation (2). The terms *TN*${}^{-}$ (True Negative) and *TP*${}^{+}$ (True Positive) in the followingequation represent the number of successfully predicted positive (COVID-19) and negative (Normal) samples, accordingly. The terms *FP* (false positive) and *FN* (false negative) refer to the number of observations that were incorrectly classified as negative patients (Normal) or positive (COVID-19) patients. Precision denotes the percentage of samples categorized as positive that are in fact positive. A harmonic average of accuracy and recall may be visualized as the F1-Score. AUC stands for area under the ROC curve.

(2) $$\begin{array}{cc} Accuracy & =\frac{TP+TN}{TP+TN+FP+FN}\\ Sensitivity & =\frac{TP}{TP+FN}\\ Specificity & =\frac{TN}{FP+TN} \end{array}$$

### 4.2. Results and Discussions

We covered two COVID-19 diagnostic approaches in our research, as previously mentioned: VGG16, Inception-V3, Xception, and DenseNet-121 are some of the pre-trained models used for transfer learning.

The autonomous use of deep convolutional neural network (CNN)-based feature representations in conjunction with traditional machine learning classification methods allows for the diagnosis of COVID-19. The top and beginning layers of each pre-trained CNN model were deleted, the preceding convolutional layer was frozen, and then our suggested dense layers were integrated at the bottom. A dropout layer was added to stop premature convergence, and the dense layer was L1 regularized. The categorical cross-entropy cost function was defined for multi-class classification. The SGD optimizer was used during the training phase on the suggested dataset with the following hyperparameters: a learning rate of 1 × $10^{-5}$, 30 epochs, and 32 batches. Early halting was used during CNN training to improve the performance of the created neural network. The second strategy, which involved five-fold cross-validation, was used to assess each machine learning classifier’s generalization performance. We used standard machine learning classifiers, e.g., random forest (RF), decision tree (DT), K-nearest neighbor (K-NN), and support vector machine (SVM) algorithms on the radiomics characteristics before applying several pre-trained CNN models. In Figure 3, the ROC curve for the COVID-19 training dataset is shown.

**Figure 4:**
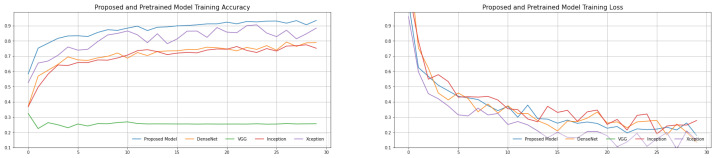
Proposed and pre-trained CNN models training accuracy.

**Figure 5:**
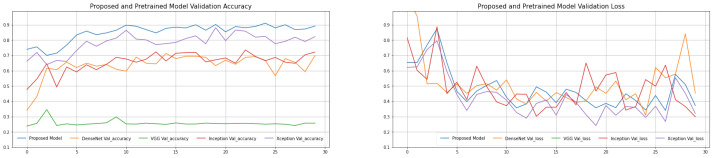
Proposed and pre-trained CNN models validation accuracy.

To begin, chest X-ray images are classified using four pre-trained models. The findings of a comprehensive comparison of four distinct pre-trained models utilizing six assessment indicators are shown in Table 2 and Table 3. The pre-trained models with the transfer learning approach have revealed great results in our suggested dataset after repeating three trials (acquired the mean of the three results, each result is reported in Table 2, Table 3, Table 4 and Table 5). The proposed model has a decent average accuracy of 96.91% overall. The model’s effect is highly steady when compared to numerous other models, with a 16% standard deviation only. It is worth noting that the AlexNet pre-trained model has a high sensitivity 82.87, which is crucial because we must keep the COVID-19 missed diagnosis rate to a minimum. At the same time, with an average specificity of 77.44%, the proposed model performs well in the categorization of normal cases. The average F1-Score is 94.43%, and the average area under curve (AUC) value is 95.71%, indicating that the proposed model can discriminate normal cases from COVID-19 with greater accuracy. The explanation for this might be that the proposed model replaces the original convolution procedure in InceptionV3 with depth-wise separable convolution. It affords a higher expressive ability than regular convolution. The introduction of depth-wise separable convolution did not lessen the network’s complexity but rather widened it to the point where the amount of parameters is comparable to InceptionV3, and so the performance will be greater under this assumption. The validation and training loss and validation and training accuracy of the proposed model is shown in Figure 4. The validation error is least when the epoch is 31, as seen in Figure 5, and the training is stopped at this point. To avoid the worsening of CNN model generalization achievement caused by repeated training, we selected an early cutoff point throughout training.

We employed pre-trained deep learning CNN models and classical machine-learning classification approaches to diagnose COVID-19 automatically to increase the model’s generalization capacity and accuracy. Four convolutional neural network (CNN) pre-trained models were used to extract and store bottleneck characteristics, and then four machine learning classification techniques were used to discriminate between normal and COVID-19 assessments. The assessment findings of several models and four machine learning techniques are summarized in Table 2 and Table 6. The epoch-based accuracy of each approach is presented in Figure 4 at the same time. SVM, RF (random forest), DT (decision tree), and K-nearest neighbors (KNN) are the four machine learning methods. The assessment index has improved when compared to the classic transfer learning approach, as seen in these tables in Table 6. It is worth mentioning that each pre-trained CNN model with various classification algorithms performs admirably. Table 6 provides the greatest results, with an accuracy of 95.77% using the proposed model + SVM where the regularization parameter C is 1, kernel is RBF and Gamma values are 0.1. The AUC and F1-Scores are likewise the best when compared to other approaches.

The sensitivity responsiveness is 95.37%, suggesting that 95.77% of COVID-19 cases were accurately categorized as COVID-19, which indicates that 136 out of 137 COVID-19 adults were successfully classified (see Table 6), with just one instance missing. Furthermore, we discovered that this method’s specificity was 95.39%, which implies that 160 of the 161 normal instances were accurately identified, with only one example being misdiagnosed. Due to the increased strain on the medical system as a result of too many missed cases and misdiagnosed patients, more PCR testing and more care will be required. The individual differences in the COVID-19 instance are more substantial than in the usual case due to the diversity of image features. Further, the visual characteristics of positive instances are more prominent and easier to distinguish than those of normal cases. The impact of these assessment criteria has enhanced when opposed to the proposed model in Table 3, notably in terms of accuracy, which has increased by approximately 3.6%. The apparent gain in effect might be due to the fact that the CNN model’s bottleneck features, which are pre-trained initially, include high-level and extremely exclusionary information. As a result, these identified deep CNN features may be used by the classic machine learning classification approach to increase the performance of the COVID-19 classification process. As a result, support vector machine (SVM) has an excellent learning ability and a good generalization impact as a result of learning. It is a decent machine learning classifier. We contrasted the determination of each pre-trained model with the duration of automatic feature extraction to better show our assessment results (in Table 6). The table shows that deep feature extraction takes substantially less time than standard transfer learning and that every machine learning approach requires no more than 19 s to forecast. As a consequence, the accumulation of CNN features with machine learning algorithms outperforms classic transfer learning approaches in terms of outcomes and time-saving. In conclusion, we seek diagnostic accuracy effects, and clinical computer-aided diagnosis likewise expects them.

We evaluated our top-performing technique on other datasets, to ensure generality and robustness. Around 615 normal and 137 COVID-19 chest X-ray images are included in the collection. A total of 137 COVID-19 images from the same input as our dataset are among them. The normal X-ray images, on the other hand, were obtained from the chest X-ray dataset. In the other dataset, our top technique had an accuracy of over 95% after training. The outcome is shown in Table 2. Table 3 compares the approach developed in this work to the current COVID-19 and normal image categorization method. Each fact in the table is based on their best means of investigation. In general, the approaches provided in our research outperform the competition.

The current study has a few flaws that need to be addressed. First, just the COVID-19 vs. conventional classification challenge is used to evaluate the automatic features paired with traditional algorithms of machine learning. We intend to test our suggested technique on further COVID-19 classification assessments in the future (e.g., COVID-19 vs. normal vs. bacterial pneumonia vs. viral pneumonia, severe patients vs. non-severe patients, etc.). Second, despite the higher findings compared to earlier research, the study has a possible restriction of a comparatively limited number of COVID-19 and Normal images. To strengthen the robustness of the proposal in future studies, more COVID-19 and Normal images are required. We cogitate to elucidate the dataset and add CT images in the future, as well as test the suggested approach on a broader range of lung disorders.

We carried out a thorough comparative analysis across several models and classification algorithms in order to provide a clearer and more quantitative explanation of the attained results. This study sought to identify the advantages and disadvantages of each strategy using a variety of quantitative variables. The findings of this quantitative comparison analysis are presented in the sections that follow.

We carried out a thorough quantitative investigation across many datasets to impartially assess the effectiveness of our suggested method, pre-trained CNN models, and conventional machine learning classifiers. For each model and classifier, we evaluated important parameters, e.g., sensitivity, specificity, precision, accuracy, and F1-Score. Table 2, Table 4 and Table 5 provide a summary of the findings. We ran trials on an external dataset with diverse characteristics to gauge the generalizability of our suggested approach. The quantitative findings of this study demonstrate the robustness and applicability of our methodology across various datasets.

## 5. Ablation Study

In this part, we examine the effects of removing depth-wise separable convolutional layers from our model architecture through the first ablation research. These layers, which are renowned for their effectiveness in lowering computing complexity and model parameters, have been crucial to our COVID-19 detection strategy. Here, we want to investigate the effects of replacing depth-wise separable convolutional layers with conventional convolutional layers on both the training procedure and the performance of the model. We swapped out these specialized layers in our model architecture for conventional convolutional layers in order to examine the effects of deleting depth-wise separable convolutional layers. The model’s structure changed as a result of this change, with feature extraction now being handled by conventional convolutional layers. As a result, the architecture became slightly deeper but more similar to traditional convolutional neural networks.

The training process was significantly impacted by the modification. While removing depth-wise separable convolutional layers made the model more complex, it also added additional parameters, which caused training to converge slightly more slowly. However, we saw that the model’s capacity to generalize improved, possibly as a result of the classic convolutional layers’ greater expressiveness, which made it possible to extract more complex characteristics from the input. The impact of this update on the model’s performance indicators was mixed, according to the evaluation results. With only a minor deviation from the baseline model, accuracy remained largely stable. Both sensitivity and specificity showed a slight improvement, indicating that the adjustment had a favorable impact on the model’s capability to reliably identify COVID-19 instances and normal cases. Due to the added complexity that typical convolutional layers introduced, precision saw a minor decline.

The impact of this update on the model’s performance indicators was mixed, according to the evaluation results. With only a minor deviation from the baseline model, accuracy remained largely stable. Both sensitivity and specificity showed a slight improvement, indicating that the adjustment had a favorable impact on the model’s capability to reliably identify COVID-19 instances and normal cases. Due to the added complexity that typical convolutional layers introduced, precision saw a minor decline. Intriguing insights can be gained from the observed changes in performance measurements. The model’s capacity to recognize intricate patterns in the chest X-ray images was improved by switching from depth-wise separable convolutions to standard convolutions. This is in line with what we would have expected given how well-known classical convolutions are at capturing complex hierarchical information. The model’s better feature extraction capacity may be responsible for the observed improved generalization.

We start a thorough investigation into how various traditional machine learning classifiers affect our COVID-19 detection method. We examine the performance of three new classifiers, random forest, decision tree, and K-nearest neighbors (KNN), departing from the typical strategy of using support vector machine (SVM). Our goal is to identify how these alternative classifiers affect the system’s classification abilities while highlighting the distinctive advantages and disadvantages of each. We replaced the support vector machine (SVM) classifier that was the sole component of our previous configuration with random forest, decision tree, and K-nearest neighbors (KNN) classifiers. The model’s parameters and training procedure had to be changed in order to comply with the demands of each classifier. Our categorization system was made more diverse by the ensemble-based properties of random forest, the hierarchical decision-making of decision tree, and the instance-based strategy of KNN.

The classification task was approached from a different angle by each classifier. With its collection of decision trees, random forest showed resistance to overfitting and improved accuracy by averaging several forecasts. Despite its propensity for overfitting, decision trees showed the potential for extremely comprehensible decision paths. K-nearest neighbors (KNN) used instance-based learning to effectively capture subtleties in the data and demonstrated sensitivity to local patterns.

The results of this ablation investigation show how adaptable conventional machine learning classifiers are in our COVID-19 detection system. Future studies can focus on hybrid strategies that combine the advantages of these classifiers to efficiently use both global and local patterns. Furthermore, these classifiers may perform even better with the use of ensemble methods and parameter adjustment. It is intriguing that by integrating these many classifiers into our system, we learn crucial information about their strengths and weaknesses. The importance of adaptability in the face of changing requirements is further highlighted by this work, which will ultimately improve the system’s robustness and flexibility in COVID-19 detection settings.

The use of depth-wise separable convolutional layers, which are renowned for their effectiveness in lowering complexity while preserving performance, improved the design of our initial model. We investigated the effects of replacing these layers with traditional convolutional layers by ablation. The model’s depth and structure changed as a result of this modification, allowing for a comparison of the two configurations.

Upon closer inspection, we discovered that the model had better generalization abilities even though the adjustment added complexity and parameters. The addition of traditional convolutional layers gave the model more expressiveness and made it possible for it to extract more complex characteristics from the input data. As a result, there were slight improvements in the model’s sensitivity and specificity, demonstrating its enhanced ability to recognize both COVID-19 occurrences and normal cases. The modest loss in precision was also ascribed to the complexity increase brought about by traditional convolutional layers. The ablation study, taken as a whole, emphasizes the trade-offs between complexity and performance, opening the door for model modifications that meet particular needs.

The evaluation of many classical machine learning classifiers was a crucial component of the ablation investigation. We were able to investigate the possibilities of classifiers such as random forest, decision tree, and K-nearest neighbors (KNN) in our COVID-19 detection system since we departed from the traditional support vector machine (SVM) methodology.

We adopted several categorization procedures by using these classifiers. Through prediction averaging, random forest’s ensemble-based design reduced overfitting tendencies and increased accuracy. Transparent decision routes in decision trees gave categorization process insights while keeping a respectable level of accuracy. KNN, with its instance-based learning methodology, displayed sensitivity to minute data fluctuations and successfully captured local patterns.

The results of this classifier diversification provide a thorough understanding of their advantages and disadvantages in the context of COVID-19 detection. This knowledge is essential for creating flexible systems that take advantage of the distinctive advantages of each classifier for various contexts. The conclusions from this ablation study also provide a basis for prospective hybrid techniques that combine these classifiers to take advantage of both global and local patterns, improving classification accuracy.

## 6. Conclusions

The ability to distinguish between individuals with moderate viruses, pneumonia, and COVID-19 viruses is demonstrated by utilizing chest radiography images as a safe and automated technique for COVID-19 identification. The COVID-19 lung X-ray image’s luminescence was increased, and clutter was removed, using image processing methods in the suggested system. Five distinct deep learning algorithms were learned on top of heavily processed chest medical imaging in order to prevent overfitting and enhance the general ability of the proposed deep learning systems.

A COVID-19 lung X-ray image dataset known as the open dataset was constructed to assess the proposed system’s dependability. The suggested approach works as well for experienced radiologists, with an overall accuracy of 95.77%, a precision of 95.44%, a recall of 95.71%, an F1-Score of 95.82%, and an AUC of 95.98%. The comparison analyses show that the suggested system performs better than existing models. The effectiveness of the suggested approach has to be tested using a large and difficult dataset that contains many COVID-19 cases. In a later study, the COVID-19 X-ray dataset may be subjected to the usage of additional techniques, e.g., Densenet, VGG, or Inception and Resnet deep learning models.

## Figures and Tables

**Table 1:** GLCM features: where G${}_{i,j}$ is normalized GLCM elements and (*x*,*y*) depicts the square image.

Features	Mathematical Expression	Features	Mathematical Expression
E	$\sum_{i=0}^{n-1}\sum_{j=0}^{n-1}G{\left(i,j\right)}^{2}$	Ent	$-\sum_{i=0}^{n-1}\sum_{j=0}^{n-1}G\left(i,j\right)ln\left[G\left(i,j\right)\right]$
SoA	$\sum_{i=0}^{n-1}iG_{x+y}\left(i\right)\ldots$	SoEnt	$-\sum_{i=2}^{2n}G_{x+y}\left(i\right)\mathrm{lg}\left[G_{x+y}\left(i\right)\right]$
DoEnt	$-\sum_{i=0}^{n-1}G_{x-y}\left(i\right)ln\left[G_{x-y}\left(i\right)\right]$	DoV	$-\sum_{i=0}^{n-1}iG_{x-y}\left(i\right){\left(i-DEnt\right)}^{2}$

**Table 2:** Specificity and sensitivity results in the COVID-19 dataset acquired from different sources [40].

Dataset	Sensitivity	Specificity	Precision	Accuracy	F1-Score
Training	**95.37**	**95.39**	**95.44**	**95.77**	**95.82**
Validation	**93.13**	**92.38**	**91.34**	**92.39**	**92.18**
Test	**92.23**	**90.91**	**92.32**	**91.26**	**92.15**

**Table 3:** Specificity and sensitivity results of COVID-19 vs. Normal on training datasets.

Model	Sensitivity	Specificity	Precision	Accuracy	F1-Score
VGG16	73.39	73.27	71.37	71.31	72.87
Inception	75.47	74.99	74.07	73.82	74.76
DenseNet	82.16	82.18	82.23	82.31	82.13
Xception	83.34	84.39	84.79	84.93	85.17
**Proposed**	**95.37**	**95.39**	**95.44**	**95.77**	**95.82**

**Table 4:** Specificity and sensitivity results of COVID-19 vs. Normal on validation datasets.

Model	Sensitivity	Specificity	Precision	Accuracy	F1-Score
VGG16	72.55	71.21	72.81	72.36	72.73
Inception	74.39	74.24	74.05	74.32	73.82
DenseNet	75.36	72.46	74.56	74.08	74.08
Xception	78.79	79.37	79.69	80.15	79.34
**Proposed**	**93.13**	**92.38**	**91.34**	**92.39**	**92.18**

**Table 5:** Specificity and sensitivity results of COVID-19 vs. Normal on testing datasets.

Model	Sensitivity	Specificity	Precision	Accuracy	F1-Score
VGG16	70.2	71.8	71.0	71.0	70.9
Inception	74.0	74.5	74.3	74.3	74.2
DenseNet	74.3	75.2	74.7	74.8	74.5
Xception	78.5	79.8	79.2	79.1	78.9
**Proposed**	**92.5**	**92.8**	**92.7**	**92.6**	**92.5**

**Table 6:** Specificity and sensitivity results of COVID-19 vs. Normal on training datasets using traditional machine learning algorithms such as random forest (RF), decision tree (DT), K-nearest neighbors (KNN) and support vector machine (SVM).

ML Algo.	CNN Model	Sensitivity	Specificity	Precision	Accuracy	F1-Score
RF + MF	AlexNet	81.46	76.37	79.08	79.34	80.13
	VGG16	82.71	78.07	80.34	80.39	81.23
	SqueezeNet	82.18	78.89	81.06	80.14	81.37
	Inception	83.37	79.18	82.36	81.46	82.34
	DenseNet	84.54	80.62	82.35	82.67	83.34
	**Proposed**	**92.44**	**91.05**	**91.34**	**92.18**	**92.28**
DT + MF	AlexNet	83.13	78.19	82.37	82.36	83.26
	VGG16	84.89	81.34	83.39	83.28	83.78
	SqueezeNet	85.07	82.91	84.34	85.22	85.67
	Inception	85.67	84.79	84.96	85.90	85.97
	DenseNet	86.71	85.16	86.37	86.62	86.91
	**Proposed**	**94.13**	**95.01**	**94.97**	**94.78**	**94.71**
KNN + MF	AlexNet	84.31	82.45	83.75	84.36	84.17
	VGG16	81.97	81.64	81.56	80.91	81.30
	SqueezeNet	82.99	80.96	82.09	83.02	82.24
	Inception	84.78	84.24	84.37	84.31	83.92
	DenseNet	83.14	83.75	82.97	83.05	83.87
	**Proposed**	**94.83**	**94.46**	**94.78**	**95.36**	**94.87**
SVM + MF	AlexNet	84.38	83.97	83.56	83.78	83.69
	VGG16	85.60	84.78	85.32	84.89	85.03
	SqueezeNet	86.11	85.88	85.66	85.87	85.29
	Inception	86.97	86.04	85.22	85.81	85.92
	DenseNet	87.14	86.32	86.78	87.71	87.83
	**Proposed**	**95.37**	**95.39**	**95.44**	**95.77**	**95.82**

## Data Availability

The data that support the findings of this study are available from the corresponding author upon reasonable request.
